# Oxidized Low-Density Lipoprotein Is a Novel Predictor of Interferon Responsiveness in Chronic Hepatitis C Infection

**DOI:** 10.1016/j.jcmgh.2015.03.002

**Published:** 2015-03-14

**Authors:** Philipp Solbach, Sandra Westhaus, Maximilian Deest, Eva Herrmann, Thomas Berg, Michael P. Manns, Sandra Ciesek, Christoph Sarrazin, Thomas von Hahn

**Affiliations:** 1 Department of Gastroenterology, Hepatology and Endocrinology, Medizinische Hochschule Hannover, Hannover, Germany; 2 German Center for Infection Research (DZIF), Hannover, Germany; 3 Institute for Molecular Biology, Medizinische Hochschule Hannover, Hannover, Germany; 4 Institute of Biostatistics and Mathematical Modeling, Johann-Wolfgang-Goethe-Universität, Frankfurt am Main, Germany; 5 Hepatology Section, Department of Gastroenterology and Rheumatology, Universitätsklinikum Leipzig, Leipzig, Germany; 6 Medical Clinic I, Zentrum der Inneren Medizin, Klinikum der Johann-Wolfgang-Goethe-Universität, Frankfurt am Main, Germany

**Keywords:** Cell-to-Cell Spread, oxLDL, SVR, SR-BI, DAA, direct-acting antiviral drug, DMEM, Dulbecco’s modified Eagle medium, DTT, dithiothreitol, HCV, hepatitis C virus, HCVcc, cell culture–grown hepatitis C virus, IPS1, interferon promoter stimulator-1, ITX-5061, *N*-[5-tert-butyl-3-(methanesulfonamido)-2-methoxyphenyl]-2-[4-(2-morpholin-4-ylethoxy)naphthalen-1-yl]-2-oxoacetamide;hydrochloride, LDL, low-density lipoprotein, NLS, nuclear localization signal, oxLDL, oxidized low-density lipoprotein, PBS, phosphate-buffered saline, peg-IFN, pegylated interferon α, RBV, ribavirin, RFP, red fluorescent protein, ROC, receiver operating characteristic, SR-BI, scavenger receptor class B member I, SVR, sustained virologic response

## Abstract

**Background & Aims:**

Hepatitis C virus (HCV) cell entry is mediated by several cell surface receptors, including scavenger receptor class B type I (SR-BI). Oxidized low density lipoprotein (oxLDL) inhibits the interaction between HCV and SR-BI in a noncompetitive manner. We tested whether serum oxLDL levels correlate with sustained virologic response (SVR) rates after interferon-based treatment of chronic hepatitis C.

**Methods:**

Baseline oxLDL was determined in 379 participants with chronic HCV genotype 1 infection from the INDIV-2 study using a commercial enzyme-linked immunosorbent assay. The mechanistic in vitro studies used full-length and subgenomic HCV genomes replicating in hepatoma cells.

**Results:**

In the multivariate analysis, oxLDL was found to be an independent predictor of SVR. Oxidized LDL did not correlate with markers of inflammation (alanine transaminase, ferritin), nor was serum oxLDL affected by exogenous interferon administration. Also, oxLDL did not alter the sensitivity of HCV replication to interferon. However, oxLDL was found to be a potent inhibitor of cell-to-cell spread of HCV between adjacent cells in vitro. It could thus reduce the rate at which new cells are infected by HCV through either the cell-free or cell-to-cell route. Finally, serum oxLDL was significantly associated with the estimated infected cell loss rate under treatment.

**Conclusions:**

Oxidized LDL is a novel predictor of SVR after interferon-based therapy and may explain the previously observed association of LDL with SVR. Rather than being a marker of activated antiviral defenses it may improve chances of SVR by limiting spread of infection to naive cells through the cell-to-cell route.

**Summary**

The unexplained association between interferon responsiveness and serum low-density lipoprotein (LDL) in chronic hepatitis C is likely due to oxidized LDL, a subfraction that blocks viral cell entry by perturbing the interaction between hepatitis C virus and its primary receptor.

The hepatitis C virus (HCV) is a small enveloped virus with a single-stranded, positive-sense RNA genome. It has chronically infected an estimated 160 million individuals worldwide and is a leading cause of end-stage liver disease. Pegylated interferon α (peg-IFN) in combination with ribavirin (RBV) had long been the standard treatment. Beginning in 2011 several direct-acting antiviral drugs (DAA) have been approved, and more are expected to follow.^1^ Currently, both peg-IFN-containing and interferon-free treatments are available.

About half of individuals infected with HCV genotype 1 achieve sustained virologic response (SVR)—clearance of infection—after treatment with peg-IFN/RBV.^2^ Numerous factors have been associated with interferon responsiveness and eventual SVR. These have been reviewed elsewhere.^3^ Notably, genetic and nongenetic factors interact to determine chances of SVR.^4^ Among the biochemical parameters, high low-density lipoprotein (LDL)^5^, ^6^, ^7^, ^8^ and low γ-glutamyltranspeptidase^9^ seem to be strongly predictive of SVR. However, for these and most other factors the mechanism of how they are linked to interferon responsiveness is unclear, so our ability to predict treatment outcome in individual patients remains unsatisfactory.

There are multiple interconnections between the HCV replication cycle and host lipid metabolism: HCV particle assembly is linked to the host cell’s very-low-density lipoprotein synthesis machinery, resulting in the release of “lipoviral particles” containing both lipoprotein and viral components.^10^, ^11^, ^12^, ^13^ Indeed, HCV particles produced in cell culture (HCVcc) appear to be less dense and larger than expected for a member of the *Flaviviridae*. Moreover, HCVcc are heterogeneous in size, and larger particles appear to be more highly infectious.^14^, ^15^ An essential HCV receptor on hepatocytes is scavenger receptor class B type I (SR-BI),^16^ physiologically the main receptor for high-density lipoprotein on hepatocytes. The LDL receptor, on the other hand, has long been proposed to also be involved in HCV cell entry,^17^ but its exact role has remained controversial, and unlike SR-BI it has not been shown to be essential for productive infection.^18^

Despite these clear links between the HCV replication cycle and both very-low-density lipoprotein and high-density lipoprotein, LDL is the lipid species most consistently associated with SVR after peg-IFN/RBV as well as first-generation telaprevir-based triple therapy.^5^, ^6^, ^7^, ^8^, ^19^ This is somewhat puzzling because the link between LDL and HCV biology is not obvious. We have previously identified and characterized oxidized low-density lipoprotein (oxLDL)—a naturally occurring derivative of native LDL that has undergone oxidative modifications—as a potent endogenous inhibitor of HCV cell entry.^20^, ^21^ In vivo oxLDL is detectable at low levels in human serum. Oxidized LDL binds to SR-BI, and our earlier work suggested that it perturbs the interaction between HCV and SR-BI in a noncompetitive manner. Moreover, we were able to show that endogenous oxLDL, like the in vitro generated oxLDL which is mostly used for experimentation, inhibits HCV infectivity. However, whether endogenous oxLDL has an impact on interferon responsiveness in the setting of chronic HVC infection is unknown. This study was undertaken to address this question.

## Materials and Methods

### Patient Cohort

We examined the samples and clinical data of 379 treatment-naive patients infected with HCV genotype 1 from the treatment arm of the INDIV-2 study. Details of the study have been described elsewhere.^22^ Briefly, the patients received peg-IFN/RBV for 24–72 weeks depending on their baseline viral load and on-treatment viral kinetics. The INDIV-2 study protocol was approved by the independent ethics committees at all 20 German study centers.

### Oxidized Low-Density Lipoprotein Enzyme-Linked Immunosorbent Assay

To quantify serum oxLDL, we used a commercial enzyme-linked immunosorbent assay system (cat. no. 10-1143-01; Mercodia, Uppsala, Sweden) that we had previously evaluated.^21^

### Cell Lines and Reagents

Huh-7 and Huh-7.5 cells were maintained in Dulbecco’s modified Eagle medium (DMEM) (cat. no. 11966-025; Life Technologies, Darmstadt, Germany) supplemented with 10% fetal calf serum (cat. no. F7524-500ML; Sigma-Aldrich, Munich, Germany), nonessential amino acids (cat. no. 11140-035; Life Technologies), l-glutamine (cat. no. 25030-024; Life Technologies), and penicillin/streptomycin (cat. no. A 2212; Biochrom, Berlin, Germany). Oxidized LDL was purchased from Intracel Resources (cat. no. RP-047; Frederick, MD) and LDL from Kalen Biomedical (cat. no. 770200-4; Montgomery Village, MD). Peg-IFN for in vitro experimentation was obtained from Roche Pharma (Basel, Switzerland).

### Plasmids

For pseudo-particle production, we used the following three plasmids: (1) a vector expressing the human immunodeficiency virus (HIV) gag and pol genes (HIV gag-pol), a plasmid encoding the G-protein of the vesicular stomatitis virus, and pTRIP-tagRFP-NLS-IPS1 encoding red fluorescent protein (RFP) fused to a nuclear localization signal (NLS) and the mitochondrial anchor domain of IPS1 (interferon promoter stimulator-1).^23^ The plasmids pFK_JFH1/J6/C-846.dg and pFK_i389luc_EL_JFH1/J6/C-846.dg encoding the full-length chimeric HCV genotype 2a genome Jc1 with or without a firefly luciferase reporter (Jc1 and Fluc-Jc1, respectively) have been described by Pietschmann et al.^24^ Moreover, subgenomic replicons containing a luciferase reporter representing genoytpes 2a (Luc-JFH1-NS3-5B) and 1b (Fluc-Con1 NS3-5B) were used.

### Production of Pseudo-particles and Transduction of Target Cells

**Supplementary Figure 1:**
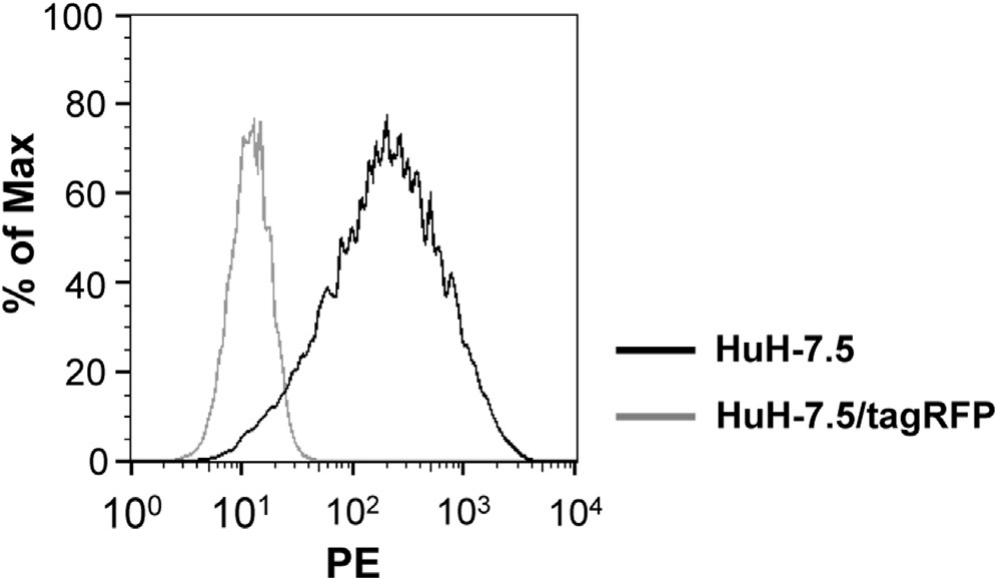
**Expression level of red fluorescence protein (RFP) in transduced Huh-7.5 cells.** Huh-7.5 cells were transduced with tagRFP-NLS-IPS1, and expression level of RFP was determined by fluorescence-activated cell sorting analysis compared with naive Huh-7.5 cells immediately before cell-to-cell spread experiments were performed.

Pseudo-particles encoding the tagRFP-NLS-IPS1 reporter were produced as previously described elsewhere.^25^ Briefly, human embryonic kidney 293T (HEK293T) cells (8 × 10^5^ cells/well) were seeded onto a six-well plate 1 day before transfection. Three plasmids expressing HIV gag-pol, tagRFP-NLS-IPS1 and the G-protein of the vesicular stomatitis virus were mixed with OptiMEM (cat. no. 31985-047; Gibco/Life Technologies, Darmstadt, Germany) and polyethylenimine (cat. no. P4707-50ML; Sigma-Aldrich) as a transfection reagent. The mixture was incubated for 20 minutes at room temperature. Seeded cells were washed once with 1x phosphate-buffered saline (PBS), and 1 mL of fresh DMEM supplemented with 3% fetal calf serum, l-glutamine and nonessential amino acids was added. After incubation, 80 μL of the transfection mixture was added per well and incubated at 37°C. The medium was changed 6 hours after transfection. The pseudo-particle–containing supernatant was harvested 48 and 72 hours after transfection and pooled. The filtered supernatant was used to transduce Huh-7.5 cells. To this end, cells were incubated for 8 hours with pseudo-particle–containing supernatant before the medium was changed. The transduction efficiency was determined by flow cytometry (Supplementary Figure 1).

### Flow Cytometry

Cell populations expressing an RFP reporter were trypsinized and resuspended with DMEM complete. The cells were washed two times with 1x PBS before being resuspended in 1x PBS to a final cell number of 2 × 10^6^ cells/mL. Cells were analyzed using a FACS Canto2 cytometer (BD Biosciences, Heidelberg, Germany).

### Hepatitis C Virus RNA Transcription

We produced HCV RNA by in vitro transcription, as described by Koutsoudakis et al.^26^ Briefly, plasmid DNA encoding full-length HCV genomes or subgenomic HCV replicons was linearized by digestion with Mlu1 (cat. no. R0198L; NEB, Frankfurt am Main, Germany) for 1 hour at 37°C. Linearized DNA was purified by phenol/chloroform extraction (cat. no. 0038.1, Roti-Phenol: Carl Roth, Karlsruhe, Germany; chloroform, cat. no. 3313.1: Carl Roth), precipitated with ethanol, and dissolved in high-performance liquid chromatography (HPCL) water (cat. no. A511.2; Carl Roth). The in vitro transcription reaction mixture contained 80 mM HEPES [4-(2-hydroxyethyl)-1-piperazineethanesulfonic acid, pH 7.5] (cat. no. HN77.1; Carl Roth) 12 mM MgCl_2_ (cat. no. HNO3.1; Carl Roth), 2 mM spermidine (cat. no. SO266-5G; Sigma-Aldrich), 40 mM dithiothreitol (DTT) (cat. no. 43915-5G; Sigma-Aldrich), a 3.125 mM concentration of each nucleoside triphosphate (cat. no. 11140957001, 11140922001, 11140965001, 11140949001; Roche, Basel, Switzerland), 1 U of RNasin/mL (cat. no. N2515; Promega, Mannheim, Germany), 0.1 μg of plasmid DNA/μL, and 0.6 U of T7 RNA polymerase/μL (cat. no. P2077; Promega). After 2 hours of incubation at 37°C, 0.3 U T7 RNA polymerase was added to the mixture and incubated for another 2 hours at 37°C. The reaction was terminated by adding 1.2 U of DNase (cat. no. M6101; Promega) for 30 minutes at 37°C. After in vitro transcription, the RNA was purified by phenol/chloroform extraction (Roti-Aqua-Phenol, cat. no. A980.2; Carl Roth), precipitated with isopropanol, and dissolved in HPCL water. The RNA content and purity was determined using NanoDrop (Thermo Scientific, Schwerte, Germany).

### Hepatitis C Virus Replication Assay and Luciferase Assay

For the replication assay, the cells were trypsinized and resuspended with DMEM complete. The cells were then washed with 1x PBS and resuspended to a final cell number of 1.5 × 10^7^/mL cells in Cytomix (120 mM KCl, 0.15 mM CaCl_2_, 10 mM K_2_HPO_4_/KH_2_PO_4_, 25 mM HEPES [4-(2-hydroxyethyl)-1-piperazineethanesulfonic acid], 2 mM EGTA, 5 mM MgCl_2_, [pH 7.6]) supplemented with 2 mM ATP (cat. no. A2383-25G; Sigma-Aldrich) and 5 mM glutathione (cat. no. G4251-5G; Sigma-Aldrich). The cells were transfected with 5 μg in vitro transcribed HCV RNA by electroporation using the Gene Pulser system (BioRad Laboratories, Munich, Germany). We transferred 400 μL of cell suspension into a cuvette with a gap width of 0.4 cm (BioRad Laboratories), and the pulse was set under the following conditions: 270 V and 960 μF. Transfected cells were transferred to 37.5 mL of fresh DMEM complete and were seeded onto a 96-well plate (2 × 10^4^ cells/well) (Sarstedt, Nümbrecht, Germany).

At 4 hours after transfection, the medium was changed to medium containing different concentrations of peg-IFN (0, 0.1, 0.3, 1, 3, 10, 30, 100 ng/mL) with or without a single IC_50_ dose of oxidized LDL. The medium was changed every 24 hours to refresh the interferon and oxLDL. Transfection was stopped 24, 48, and 72 hours after transfection, and the luciferase activity was measured. Briefly, the transfected cells were washed one time with 1x PBS and lyzed directly on the plate with 35 μL of ice-cold lysis buffer (0.1% Triton X-100, 25 mM glycylglycine, 15 mM MgSO4, 4 mM EGTA, and 1 mM DTT; pH 7.8) and frozen. We then transferred 20 μL of the thawed and resuspended cells to a white 96-well plate (Lumitrac 600; Sigma-Aldrich). We measured the luciferase activity using a Luminometer Model 9100-002 (Turner BioSystems, Sunnyvale, CA) after the addition of 72 μL of assay buffer (25 mM glycylglycine, 15 mM MgSO_4_, 4 mM EGTA, 1 mM DTT, 2 mM ATP, 15 mM K_2_PO_4_, pH 7.8) and 42 μL of luciferin solution (200 μM luciferin, 25 mM glycylglycine, pH 8.0). The 50% inhibitory concentration of peg-IFN in the presence or absence of oxLDL was determined using GraphPad Prism 5 (GraphPad Software, La Jolla, CA).

### Cell-to-Cell-Spread Assay

To assay the HCVcc spread through the cell-to-cell route, we performed a modified version of the agarose overlay assay previously described elsewhere.^27^ Briefly, target Huh-7.5 cells were transduced with a tagRFP-NLS-IPS1 reporter construct.^23^ The encoded red fluorescent protein (tagRFP) fusion protein is tethered to the mitochondrial membrane in uninfected cells resulting in a perinuclear staining pattern. Upon HCV infection, the reporter fusion protein is cleaved from its mitochondrial anchor by the HCV NS3/4A protease, and due to a nuclear localization sequence it relocates to the nucleus, resulting in nuclear staining.

Huh-7.5 cells were transfected with Jc1 HCV RNA (donor cells) and incubated for 24 hours. Donor cells were mixed with naive target cells at a 1:1 ratio and seeded into 24-well plates. As soon as cells had settled (after 4 hours), the cell mixture was overlaid with medium (DMEM complete) containing 2% SeaPlaque low-melting-temperature agarose (cat. no. 50101; Lonza, Cologne, Germany) to block the virus spread through the cell-free route. The overlay was supplemented with a IC_90_ dose of oxLDL or native LDL as control. Cells were incubated for 96 hours at 37°C. The cell-to-cell spread of HCV to target cells was monitored by fluorescent microscopy (DMI6000; Leica, Wetzlar, Germany). After 96 hours of coculture, the percentage of HCV-positive target cells was determined.

### Software and Statistical Analyses

Statistical analyses of clinical data were performed with the SPSS software package (IBM, Armonk, NY). The Mann-Whitney *U*-test, Spearman correlation, receiver operating characteristic (ROC) analysis to determine the area under the ROC curve values and cutoff points, DeLong test, Fisher’s exact test, and chi-square test were performed as appropriate. A standard viral kinetic model was used to estimate the viral kinetic parameters and fit viral kinetics. Based on a maximum likelihood approach, we estimated the infected cell loss δ and mean drug efficacy using reasonable fixed values for other viral and pharmacokinetic parameters.^28^

All in vitro experiments were repeated on three or more separate occasions. Numerical data from in vitro experimentation were analyzed using an unpaired two sided *t*-test in Excel (Microsoft, Redmond, WA), and GraphPad Prism 5 was used to fit the sigmoidal concentration-response curves.

## Results

### Oxidized Low-Density Lipoprotein Is an Independent Predictor of Sustained Virologic Response

**Figure 1:**
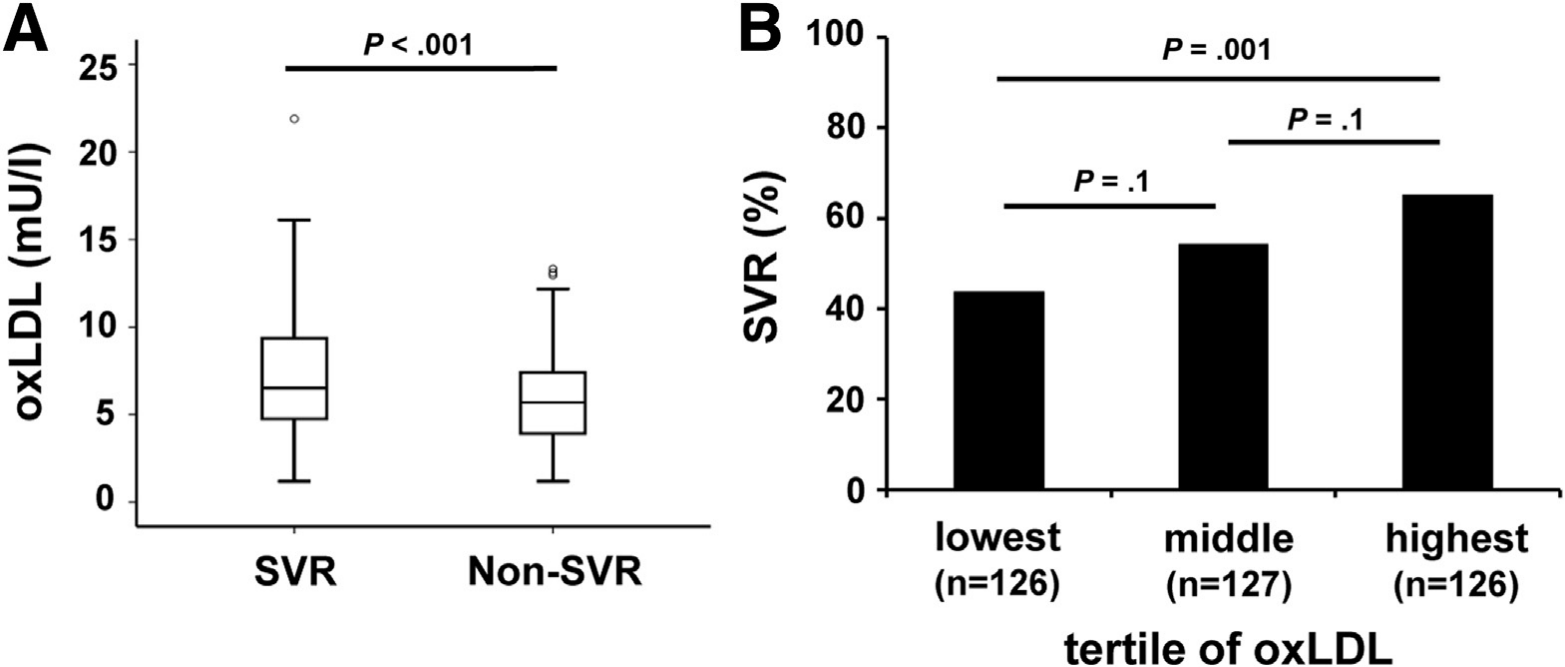
**Baseline serum oxidized low-density lipoprotein (oxLDL) is associated with sustained virologic response (SVR).** Serum oxLDL levels of 379 patient sera from the INDIV-2 cohort were determined by commercial oxLDL enzyme-linked immunosorbent assay. (*A*) Serum oxLDL in individuals (n = 206) who achieved SVR compared with those who did not (n = 173; Non-SVR). (*B*) SVR rate in patient subgroups with low, intermediate, and high baseline oxLDL. *P* values were determined by Student unpaired *t* test.

Serum oxLDL levels at baseline (ie, before treatment was started) were determined in 379 chronically HCV genotype 1 infected individuals from the INDIV-2 study.^22^ All had subsequently received treatment with peg-IFN/RBV. The serum oxLDL was statistically significantly higher in those who achieved SVR compared with those who did not (7.1 mU/L vs 5.9 mU/L; *P* < 0.001) (Figure 1*A*). Conversely, when patients were grouped into three-thirds with low, medium, and high serum oxLDL at baseline, the SVR rates were higher in groups with higher serum oxLDL (44%, 54%, and 65% in the low, intermediate, and high oxLDL groups) although only the comparison between the low and high oxLDL group reached statistical significance (Figure 1*B*).

In a univariate analysis, both LDL and oxLDL as well as other known predictors of treatment response were significantly associated with SVR (Table 1). Upon multivariate analysis, oxLDL but not native LDL remained as an independent predictor of SVR. In a ROC curve analysis, the area under the ROC curve values for oxLDL and LDL were not statistically significantly different (DeLong test, *P* = 0.82) with 0.608 (95% confidence interval [CI], 0.55–0.67) and 0.615 (95% CI, 0.56–0.67), respectively. This indicates that as a clinical test both parameters are largely equivalent. Optimal cutoff points in our cohort were determined to be 8.85 mU/L for oxLDL and 3.6 mmol/L for LDL.

### Oxidized Low-Density Lipoprotein Correlates With Native Low-Density Lipoprotein but Not With Markers of Inflammation

**Figure 2:**
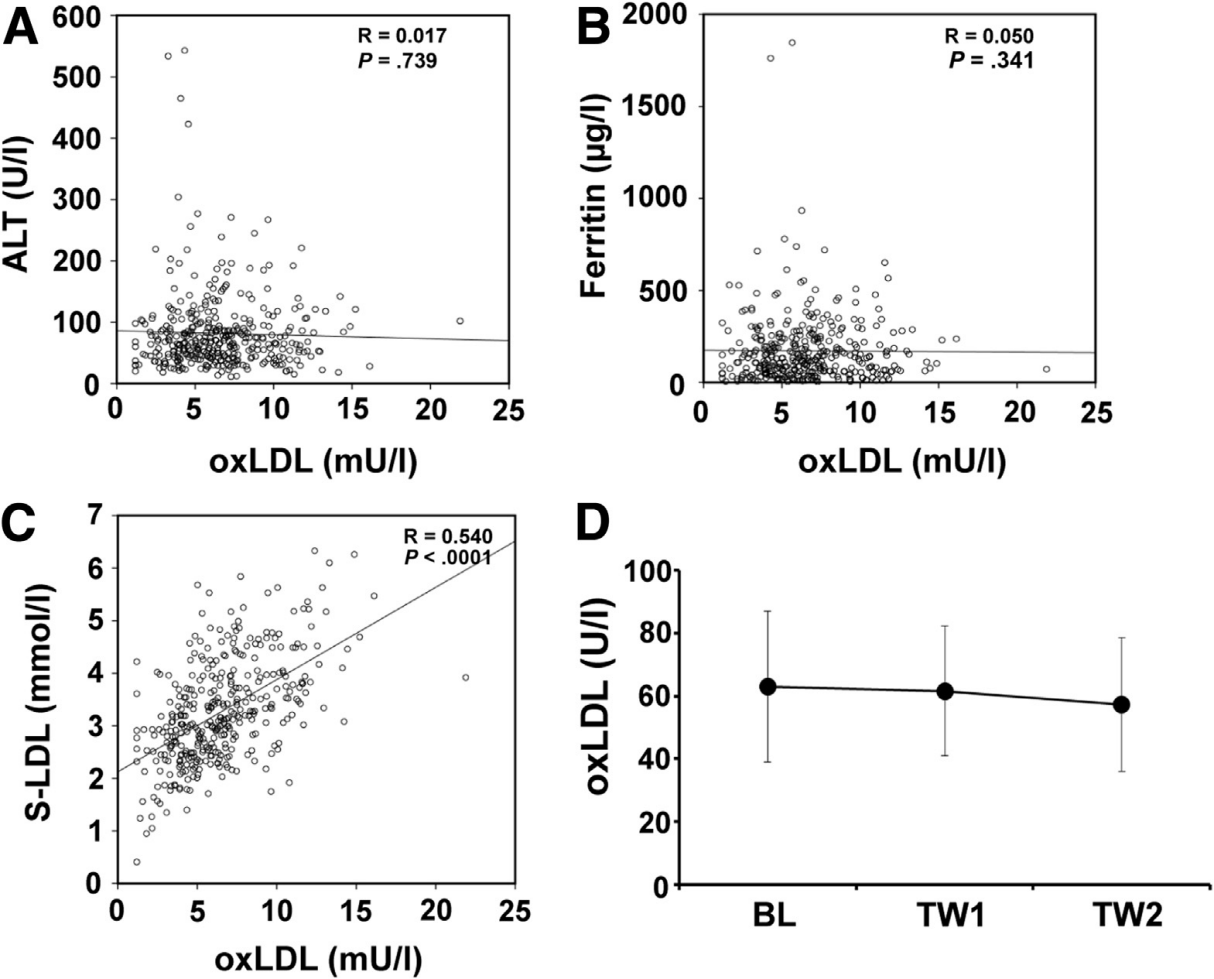
**Oxidized low-density lipoprotein (oxLDL) correlates with native LDL but not with markers of inflammation and is unaffected by interferon administration.** Baseline serum oxLDL plotted against baseline (*A*) alanine transaminase, (*B*) ferritin, and (*C*) native LDL. R describes the Spearman rank correlation. (*D*) In 26 patients serum oxLDL level were determined at baseline (BL) as well as week 1 (TW1) and 2 (TW2) of pegylated interferon α/ribavirin (peg-IFN/RBV). Data are shown as mean ± standard deviation for the respective groups.

There are several conceivable explanations for the association of oxLDL with SVR. One hypothesis was that elevated oxLDL may be associated with inflammation and as such might indicate an already ongoing antiviral response. However, there was no correlation between serum oxLDL and alanine transaminase as a marker of hepatic inflammation (Figure 2*A*) or ferritin as a marker of systemic inflammation (see Figure 2*B*). There was, however, a clear and significant correlation between serum oxLDL and native LDL (see Figure 2*C*).

### Oxidized Low-Density Lipoprotein Serum Levels Are Not Affected by Interferon

Alternatively, generation of oxLDL might be enhanced under conditions of interferon stimulation, thus it would be an indicator of activated innate antiviral defenses. We examined oxLDL serum levels during the first 2 weeks of peg-IFN/RBV administration in 26 patients from the INDIV-2 cohort. Overall, serum oxLDL remained unchanged (see Figure 2*D*). When subgroups with SVR, null response, or relapse were examined, there was also no discernible change (data not shown). Thus, serum oxLDL level appears independent of interferon stimulation.

### Oxidized Low-Density Lipoprotein Does Not Alter Interferon-Sensitivity of Hepatitis C Virus

**Figure 3:**
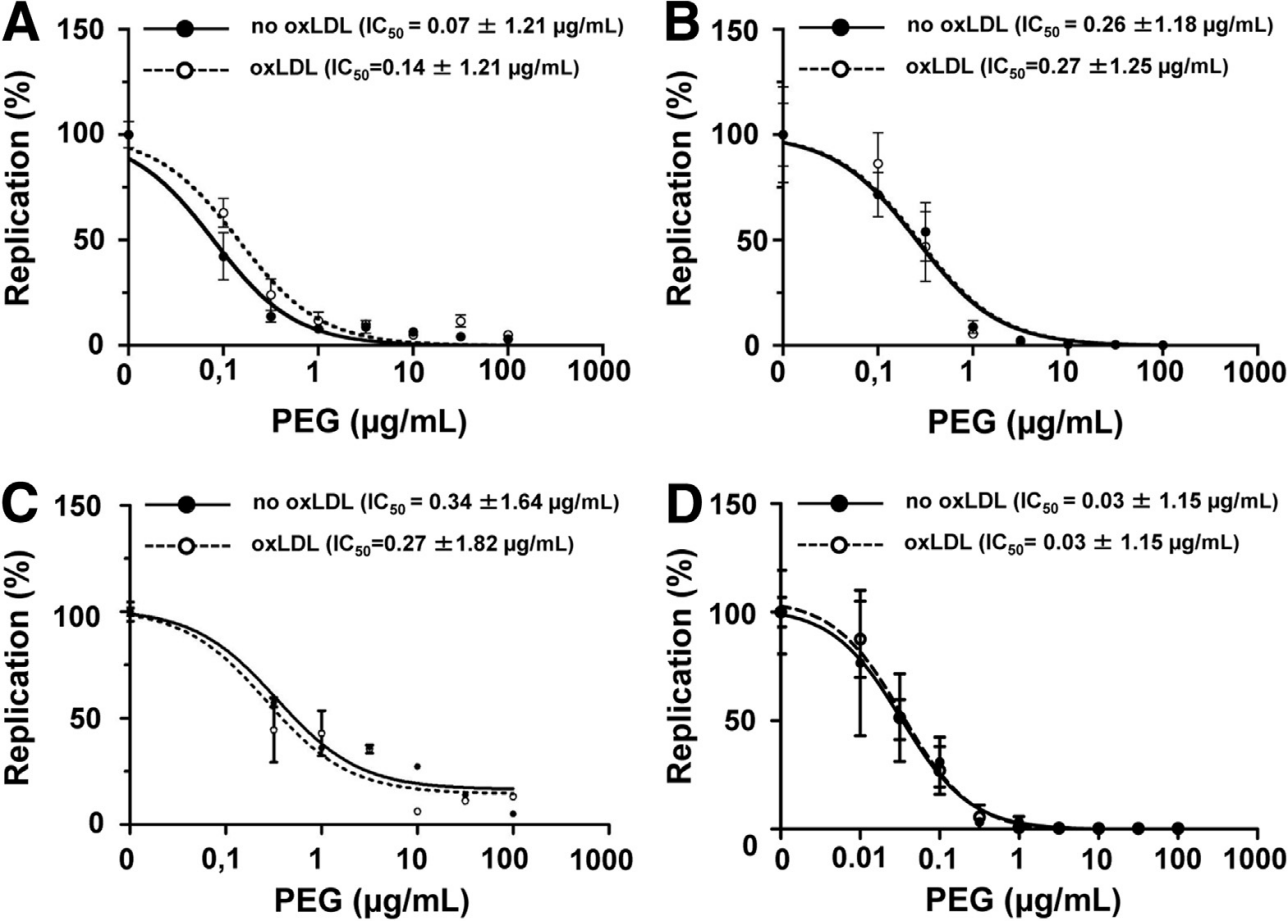
**Oxidized LDL (oxLDL) does not alter interferon sensitivity of replicating hepatitis C virus (HCV) genomes in vitro.** Huh-7.5 cells were transfected either with HCV RNA encoding (*A*) a genotype 1b NS3-5B subgenome (isolate Con1), (*B*) a genotype 2a NS3-5B subgenome (isolate JFH1), (*C*) a genotype 2a full-length genome (Jc1 chimera) all containing a firefly luciferase reporter in a separate cistron. (*D*) Huh-7 were transfected with full-length Jc1 with a firefly luciferase reporter. In all cases, the cells were treated with increasing concentrations of pegylated interferon α (peg-IFN) in the presence or absence of 10 μg/mL oxLDL. Luciferase activity was determined as a measure of RNA replication. Individual data points represent the mean ± standard deviation from a representative of three independent experiments performed in triplicate.

A second hypothesis was that oxLDL enhances the antiviral effect of exogenous interferon on HCV replication. To test this, we determined the concentration response curves for HCV inhibition by peg-IFN in the presence and absence of oxLDL (10 μg/mL). For subgenomic replicons representing genotypes 1b (Con1 isolate; Figure 3*A*) and 2a (JFH1; see Figure 3*B*) and for a full-length genotype 2a genome (Jc1; see Figure 3*C*), the IC_50_ was similar in the presence or absence of oxLDL. Similar results were observed when a full-length genotype 2a genome replication was analyzed in Huh-7 cells that have more intact innate antiviral defenses compared with Huh-7.5 (see Figure 3*D*). Thus, oxLDL does not alter interferon-sensitivity of HCV replication in this experimental system.

### Oxidized Low-Density Lipoprotein Is a Potent Inhibitor of the Cell-to-Cell Spread of Hepatitis C Virus

**Figure 4:**
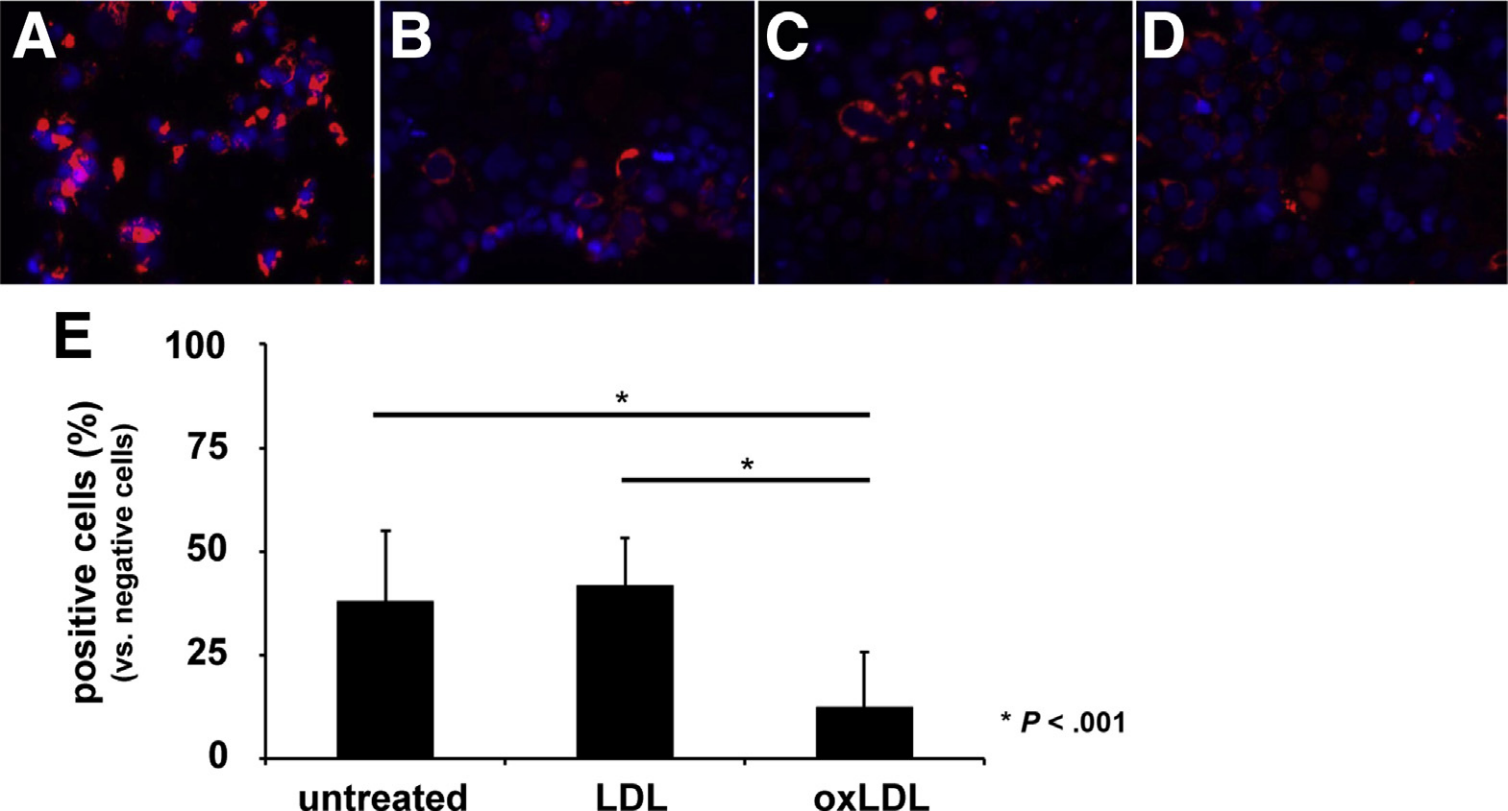
**Oxidized LDL (oxLDL) is an inhibitor of hepatitis C virus (HCV) cell-to-cell spread.** Inhibition of cell-to-cell spread by oxLDL was analyzed by coculturing unmarked HCV-infected Huh-7.5 donor cells with HCV-naive Huh-7.5 target cells expressing a tagRFP-NLS-IPS1 reporter (red fluorescence) (see Materials and Methods for details). Nuclei were stained with 4′,6-diamidino-2-phenylindole. Nuclear red fluorescence indicates infected target cells, and a mitochondrial staining pattern indicates uninfected target cells. Representative images are shown from an experiment where (*A*) mock electroporated donor cells were used and from experiments where donor cells had been electroporated with full-length HCV genomes and where seeded in the (*B*) absence or (*C*) presence of LDL (10 μg/mL) or (*D*) in the presence of oxLDL (10 μg/mL). (*E*) Results from the experimental conditions shown in *B*–*D* were quantified by counting noninfected and infected target cells and determining the percentage infected after 96 hours of coculture. Data represent the mean ± standard deviation from a representative experiment performed in triplicate counting five visual fields per well with 40–188 (mean 94 ± 43) reporter cells per visual field.

A third hypothesis was that oxLDL may improve treatment outcome by reducing infection of new hepatocytes during peg-IFN/RBV treatment, resulting in a faster decline in the pool of infected cells. Spread to uninfected cells in the setting of established chronic infection is thought to occur to a large extent through the cell-to-cell route. The effect of oxLDL on cell-to-cell spread is unknown. In an experiment where HCV-infected donor cells were mixed with uninfected acceptor cells, and spread through the cell-free route was prevented with an agarose overlay, we found that oxLDL potently inhibited cell-to-cell spread between adjacent cells with 37.8%, 41.8%, and 12.6% of acceptor cells becoming infected in the untreated, native LDL, and oxLDL treated groups, respectively (Figure 4*A*–*E*).

### Serum Oxidized Low-Density Lipoprotein Is Associated With Second Slope Decline in Viral Load

**Figure 5:**
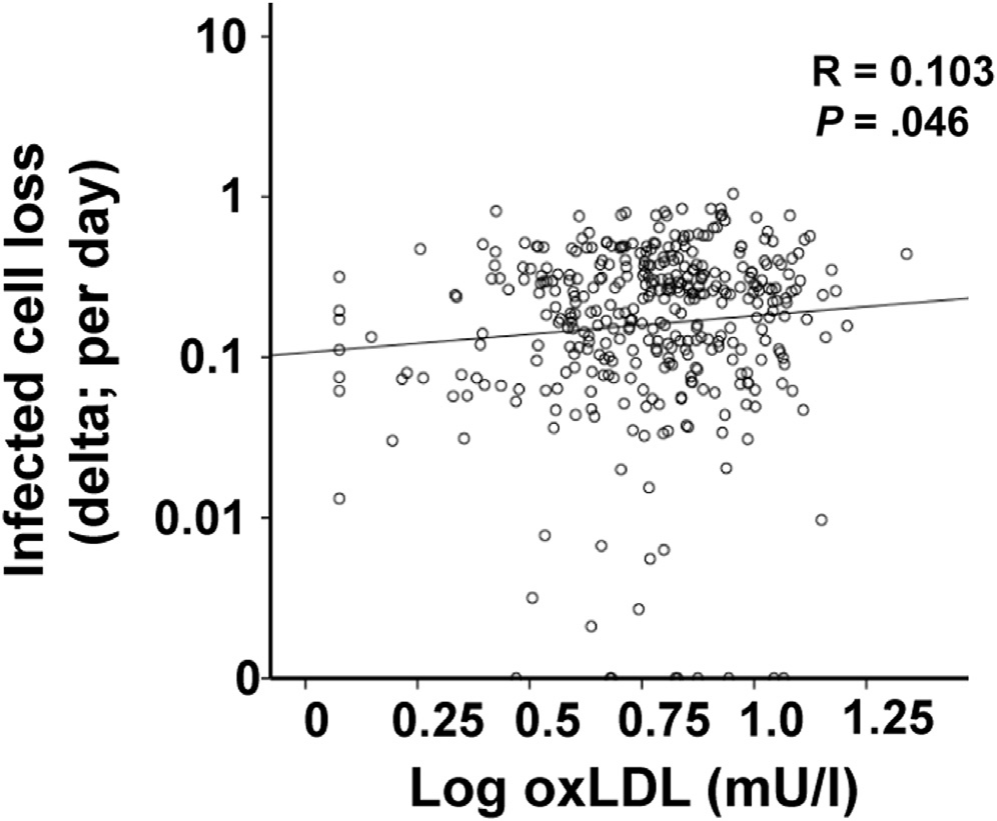
**Modest yet significant correlation of baseline serum oxidized low-density lipoprotein (oxLDL) with the estimated infected cell loss rate δ.** Correlation of baseline serum oxLDL with the estimated infected cell loss rate δ. R describes the Spearman rank correlation.

In interferon-based treatment of chronic HCV infection, decline of viral load during the second phase is thought to be driven by the rate of infected cell loss (δ), which is an important predictor for treatment response. Thus, we analyzed whether oxLDL is correlated with δ. We used viral kinetics data available from the INDIV-2 study to correlate this estimated viral kinetic parameter with serum oxLDL and found a modest yet significant correlation (Figure 5).

## Discussion

In this work, we show that baseline oxLDL is an independent predictor of response to peg-IFN/RBV treatment of chronic HCV genotype 1 infection. We provide data showing that oxLDL is not a marker of inflammation or a modulator of interferon responsiveness per se, but is a potent inhibitor of HCV cell-to-cell spread. Thus, conceivably oxLDL might enhance the likelihood of treatment response by limiting the rate at which new cells are infected during treatment.

From a clinical point of view, we have identified a novel predictor of SVR after interferon-based treatment of chronic hepatitis C. Although interferon-free regimens now offer higher SVR rates and fewer side effects compared with interferon-containing regimens, they are also much more costly.^1^ Thus, peg-IFN may continue to be used for some time, especially in settings where cost is an issue. An ability to predict who will likely achieve SVR with an interferon-based regimen and who is likely to require a more costly interferon-free regimen is thus clinically useful. However, because serum LDL is widely available as a clinical test and correlates strongly with oxLDL, it is both reasonable and cost-effective to determine LDL instead of oxLDL as a predictor for therapy outcome in the clinical setting, given that the AUC for both tests is very similar. Whether oxLDL also predicts SVR in the setting of DAA-containing regimens remains to be established. However, it has been reported that baseline LDL is associated with SVR in patients receiving triple therapy with peg-IFN/RBV plus the first generation NS3/4A protease inhibitor telaprevir.^19^ This may suggest that in this regimen oxLDL would also be predictive, given that in our cohort oxLDL correlates with native LDL but only oxLDL remains as an independent predictor after multivariate analysis. Indeed, several previous studies had observed a correlation of baseline LDL with SVR,^5^, ^6^, ^7^, ^8^ but there was no obvious explanation for this because LDL is not known to directly affect the HCV replication cycle. Our data suggest that these observations may have been secondary to the correlation of LDL with oxLDL.

We tested three different hypotheses as to why oxLDL might predict SVR. First, it has been suggested that oxLDL may be associated with the grade of inflammation in individuals with HCV infection.^29^ It has also been linked to systemic inflammation.^30^ Although to our knowledge no direct link to antiviral responses has been established, oxLDL could be a marker of inflammation or even an ongoing antiviral response and thus could predict the chances of achieving SVR through administration of exogenous interferon. However, oxLDL neither correlates with alanine transaminase as a marker of hepatic inflammation nor with ferritin, a well-known acute phase reactant. Of note, ferritin is also elevated in iron overload and advanced hepatic fibrosis among patients with nonalcoholic fatty liver disease.^31^, ^32^ Serum oxLDL itself was not affected by administration of exogenous interferon, suggesting that its serum levels are largely independent of the activation of the innate antiviral defences. Second, oxLDL might increase the sensitivity of replicating HCV to interferon. However, using state-of-the-art in vitro models of HCV infection, that is, subgenomic and full-length genomes of HCV genotypes 1 and 2 replicating in Huh-7 or Huh-7.5 cells, the concentration–response curve of peg-IFN inhibition of HCV replication was not altered in the presence of oxLDL, arguing against this explanation.

A third hypothesis is that endogenous oxLDL has a beneficial effect in the setting of peg-IFN/RBV treatment because as an HCV entry inhibitor it inhibits the infection of previously uninfected cells which in part drives infected cell loss during the untreated steady state. The pool of infected hepatocytes is a function of the rate at which infected cells disappear through cure or removal and the rate at which naive cells get infected.^33^, ^34^ The slower second phase decline of viral load during interferon-based therapy is thought to reflect a declining pool of infected hepatocytes; and the slope of second-phase decline and even more the estimated viral kinetic parameter describing the infected cell loss rate are predictive of eventual SVR. Presence of an agent reducing the rate at which naive cells get infected might thus be beneficial. We found that oxLDL but not LDL potently inhibits cell-to-cell spread between neighboring cells. Cell-to-cell spread is thought to be the dominant route of new cell infection within the chronically infected liver.^35^, ^36^ Certainly, this does not prove the hypothesis that serum oxLDL promotes viral clearance through acceleration of the decline of the pool of infected cells, but it is at least consistent with it. We also observed a modest yet significant correlation between baseline serum oxLDL and the estimated infected cell loss rate. Again, this is not proof but a finding that is consistent with a reduced rate of new cell infection. Finally, our observations that oxLDL inhibits cell-to-cell spread together with previously published data suggesting that oxLDL inhibits HCV cell entry by perturbing the interaction between the virus and SR-BI^21^ seem to suggest that HCV uses SR-BI for cell-to-cell spread as well as for infection through the cell-free route.^37^, ^38^

Whether inhibition of viral entry is a useful therapeutic approach in the setting of established chronic infection is unclear: a recent phase I trial of ITX-5061 (*N*-[5-tert-butyl-3-(methanesulfonamido)-2-methoxyphenyl]-2-[4-(2-morpholin-4-ylethoxy)naphthalen-1-yl]-2-oxoacetamide;hydrochloride), the most advanced inhibitor of HCV entry in clinical development, reported no significant effect of the drug on viral load in monotherapy.^39^ However, the effect of ITX-5061 on HCV entry is modest even in vitro.^40^ Erlotinib, a drug already approved for antitumor therapy that inhibits epidermal growth factor receptor signaling and thereby HCV cell entry,^41^ is only just beginning clinical evaluation for HCV infection. However, if cell entry inhibition had indeed a positive impact on the efficacy of interferon treatment in chronic viral hepatitis C, this might have implication for other forms of chronic viral hepatitis as well. Our data suggest that a combination of interferon with inhibitors of viral entry holds promise even in the setting of established chronic hepatitis. However, data from treatment studies will be needed to clarify the true potential of such an approach.

In summary, our work has several implications. (1) Even as all oral regimens begin to reach the market, interferon-based treatment will continue to be used in settings with cost restraints. Thus, there is a need for predictors of who is likely to respond to interferon and who will require a more expensive DAA combination regimen. (2) We show that oxLDL very likely explains why several previous studies found an association between native LDL and SVR. (3) Our data support that SR-BI is required for HCV cell-to-cell spread and that this route can be blocked by oxLDL. (4) Most importantly, we provide data suggesting that oxLDL is neither a marker of inflammation nor a modulator of the interferon response but might enhance SVR rates by limiting the rate of viral spread to uninfected cells. This finding may even be relevant for other forms of chronic viral disease as it suggests that entry inhibition may have a beneficial effect even in the context of already established infections.

## Figures and Tables

**Table 1:** Predictors of Sustained Virologic Response (SVR): Factors at Baseline That Were Associated With SVR Identified by Univariate and Multivariate Analysis

Clinical Parameter at Baseline	SVR		*P* value	
	Positive (n = 206)^a^	Negative (n = 173)^a^	Univariate Analysis	Multivariate Analysis
Age (y)	41.8 ± 10.7 (18–68)	44.9 ± 11.0 (19–68)	<.001	.1
oxLDL (mU/L)	7.1 ± 3.2 (1.2–21.9)	5.9 ± 2.6 (1.2–13.3)	<.001	<.001
Total cholesterol (mmol/L)	5.1 ± 1.2 (2.8–9.0)	4.7 ± 1.1 (1.5–8.3)	<.002	.2
HDL (mmol/L)	1.1 ± 0.4 (0.4–2.5)	1.1 ± 0.4 (0.4–3.0)	.09	.7
LDL (mmol/L)	3.5 ± 1.0 (1.5–6.3)	3.1 ± 0.9 (0.4–6.1)	<.001	.2
Triglycerides (mmol/L)	1.1 ± 0.5 (0.4–3.9)	1.1 ± 0.5 (0.2–3.6)	.8	.2
GGT (U/L)	49.2 ± 50.6 (6.0–431.0)	88.5 ± 82.3 (10.0–627.0)	<.001	.03
ALT (U/L)	80.2 ± 53.3 (11.0–357.0)	82.1 ± 59.0 (18.0–450.0)	.8	.004
ALP (U/L)	66.3 ± 18.6 (33.0–181.0)	74.0 ± 23.7 (32.0–204.0)	<.002	.007
Bilirubin (mg/dL)	1.5 ± 3.5 (0.2–37.0)	1.7 ± 3.2 (0.2–22.0)	.3	.3
Ferritin (μg/L)	133.4 ± 122.6 (3.7–780.0)	217.1 ± 237.9 (6.0–18,46.0)	<.001	<.001
Platelets (Tsd/μL)	242.6 ± 57.4 (123.0–445.0)	236.6 ± 66.9 (69.0–433.0)	.7	.9
HCV RNA (IU/mL)	975,207 ± 1,340,103 (615–7,575,860)	1,116,731 ± 1,384,396 (615–7,692,310)	<.003	.02
IL28B rs12979860, C allele present/absent (no data)	170/24 (12)^b^	126/34 (13)^b^	<.002	.003

ALT, alanine transaminase; ALP, alkaline phosphatase; GGT, γ-glutamyltranspeptidase.

a Mean ± standard deviation with range in parentheses.

b Denotes number of individuals.
